# Cardiac myocyte-specific transgenic ecSOD targets mitochondria to protect against Ca^2+^ induced permeability transition

**DOI:** 10.3389/fphys.2013.00295

**Published:** 2013-10-29

**Authors:** Jianzhu Luo, Detlef Obal, Neviana Dimova, Xian-Liang Tang, Gregg Rokosh

**Affiliations:** ^1^Division of Cardiovascular Medicine, Institute of Molecular Cardiology, University of Louisville, Louisville, KY, USA; ^2^Department of Anesthesiology and Perioperative Medicine, University of Louisville, Louisville, KY, USA; ^3^Department of Physiology and Biophysics, University of Louisville, Louisville, KY, USA

**Keywords:** ecSOD, mitochondria, cardiac myocytes, reactive oxygen species, nitric oxide

## Abstract

ecSOD function has prototypically been associated with the extracellular space due to its secretion and localization to the extracellular matrix. A myocyte-specific ecSOD transgenic mouse has shown that it can also be localized to the myocyte intracellular compartment and is capable of attenuating Reactive oxygen species (ROS) formation and increasing NO bioavailability after ischemia reperfusion. Here, the subcellular localization of transgenic ecSOD was further defined by subcellular fractionation, immunofluorescent confocal microscopy, and Western analysis. Its impact on mitochondrial function was assessed by mitochondrial permeability transition (MPT). ecSOD was found to exist in cytosolic and nuclear fractions in addition to membrane. Colocalization of ecSOD with myocardial mitochondria was further demonstrated by confocal microscopy and subcellular fractionation of mitochondria and Western analysis. Isolated ventricular myocytes from cardiac-specific transgenic ecSOD mice were protected from hypoxia reoxygenation injury. Increased ecSOD colocalization to myocardial mitochondria in ecSOD Tg hearts limited MPT in response to Ca^2+^ challenge. These results demonstrate that ecSOD is not restricted to the extracellular space and can alter MPT response to Ca^2+^ suggesting mitochondrial localization of ecSOD can affect key mitochondrial functions such as MPT which are integral to cell survival.

## Introduction

Reactive oxygen species (ROS), including superoxide anion (O^−^_2_), are an ubiquitous product of several cellular processes. ROS are important in signal transduction through the activation of NADPH oxidases and by several other mechanisms (Griendling et al., 1994) including, xanthine oxidase (Ekelund et al., 1999), nitric oxide synthase (Xia et al., 1998), a product of cellular metabolism, and may be increased as a consequence of myocardial ischemia reperfusion, with tissue injury, and other pathologies (Sawyer et al., 2002). Uncontrolled ROS formation results in oxidative tissue damage that results in progressive dysfunction and eventual cell death. A broad range of proteins exist to buffer the oxidative stress of ROS, of which, the trio of superoxide dismutases (SOD), exist to specifically degrade the highly reactive radical, superoxide. Three SODs, extracellular (ecSOD), manganese (MnSOD), and copper-zinc (CuZnSOD) are present in cells to varying degrees and differentially localized to specific compartments that orchestrate the dismutation of O^−^_2_ to hydrogen peroxide (H_2_O_2_) and O_2_.

Extracellular SOD, as one of the family of SOD enzymes, converts O^−^_2_ to H_2_O_2_ and has been shown to protect cells against the effects of this oxygen free radical. ecSOD, as its name implies, has been characterized as a secreted protein that binds to the extracellular matrix by its heparin binding domain (Sandstrom et al., 1993; Oury et al., 1994). In the myocardium, ecSOD is expressed at much lower levels with MnSOD and CuZnSOD accounting for over 90% of total SOD activity (Obal et al., 2012). Targeted deletion of ecSOD has shown that it contributes to protect in the response to doxorubicin induced myocardial injury (Kliment et al., 2009) and infarction (van Deel et al., 2008). We have also shown that ecSOD gene therapy ameliorates myocardial ischemia reperfusion injury (Li et al., 1998, 2001). Here it has been shown to protect cells from oxidative stress. Recently, we described a cardiac-specific ecSOD transgenic mouse that provides increased O^−^_2_ dismutation that subsequently supports increased NO bioavailability (Obal et al., 2012). Notably, ecSOD expression in this mouse was found to be elevated in both extracellular and intracellular compartments. Here we extend these findings by further characterizing the intracellular localization of ecSOD and demonstrate the functional consequences of mitochondrial ecSOD.

## Materials and methods

### Cardiomyocyte-specific ecSOD transgenic mice

All procedures were conducted under the approval of the University of Louisville IACUC in accordance with the NIH Guide for the Care and Use of Laboratory Animals [DHHS publication No. (NIH) 85-23, rev. 1996] (Dai et al., 2010). Cardiac-specific ecSOD Tg mice heterozygous for the transgene used in experiments were produced by crossing with WT C57BL/6 mice with littermate WT mice used as controls as previously described (Obal et al., 2012).

### Cardiac myocyte isolation for hypoxia reoxygenation injury

Adult cardiac myocytes from WT and ecSOD Tg mice were isolated as previously described and ~ 2 × 10^5^ myocytes plated per well in 24-well culture plates with inserts (NUNC) (Hu et al., 2007). After equilibration in myocyte culture media, the culture medium was immediately changed to oxygen depleted (bubbled with N_2_ for 10 min) hypoxia buffer (NaCl 118 mM, NaHCO_3_ 24 mM, NaH_2_PO_4_ 1 mM, CaCl_2_ 2.5 mM, MgCl_2_ 1.2 mM, sodium lactate 20 mM, KCl 16 mM, 2-deoxyglucose 10 mM, pH 6.4) and myocytes maintained under hypoxic conditions, 1% O_2_, 5% CO_2_, and 94% N_2_, for 30 min in a hypoxia chamber (Billups-Rothenberg). Myocytes were then reoxygenated when the hypoxia buffer was replaced with normoxic culture media (Hu et al., 2007). Myocytes were reoxygenated for 120 min. Lactate dehydrogenase (LDH) activity in media collected from myocyte culture media immediately after 120 min in normoxic buffer under normoxia was detected by colorimetric assay by measuring the formazan product at 492 nm as previously described (Roche Applied Science) (Hu et al., 2007). Total LDH release was determined in media after treating cells with 2% triton X100. LDH release in each well was expressed as a percentage of total LDH normalized to protein content determined by Bradford assay.

### Mitochondria isolation and MPT measurement

Mitochondria from WT and ecSOD hearts were isolated as previously described (West et al., 2008). WT and ecSOD hearts were minced and homogenized in buffer A, 225 mM mannitol, 70 mM sucrose, 5 mM MOPS, 2 mM EGTA, and 0.2% fatty acid-free BSA, using 5 strokes of a glass-Col homogenizer. The homogenate was centrifuged at 500×*g* for 10 min at 4°C and the supernatant filtered through cheesecloth. Mitochondria were obtained after centrifugation at 5000×*g* for 10 min at 4°C followed by 2 washes. Mitochondria (150–200 μg protein/assay) were then resuspended in 200 μl swelling assay buffer, 40 mM HEPES, 195 mM mannitol, 25 mM sucrose, and 0.01 mM EGTA for mitochondrial permeability transition (MPT) studies.

MPT activation was determined by measuring the change in optical density with Ca^2+^-induced mitochondrial swelling at 520 nm as previously described (West et al., 2008). Mitochondria were loaded into wells of a 96 well plate with 1 μM rotenone and 5 mM succinate and read at 37°C for 30 min. Mitochondria from WT and ecSOD Tg hearts were also treated with cyclosporine A (CsA, 10μM) as a control. Mitochondrial swelling was initiated with the injection of Ca^2+^ into each corresponding well with shaking after recording 5 min of baseline. Swelling was recorded for 20 min.

### Myocyte subcellular fractionation and extraction

Subcellular fractions were extracted from isolated WT and ecSOD myocytes using the ProtoExtract Subcellular Proteome Extraction kit (Calbiochem). Myocytes from WT and ecSOD Tg mice were homogenized in Fraction I buffer and gently agitated for 10 min at 4°C and centrifuged at 1000×*g* for 10 min. The supernatant was removed and stored on ice (Fraction I, cytosolic). Extraction buffer II with protease inhibitor cocktail was added to the pellet and the pellet resuspended and incubated for 30 min at 4°C with gentle agitation. The suspension was centrifuged at 6000×*g* for 10 min at 4°C and the supernatant collected (Fraction II, membrane) on ice. Extraction buffer III with protease inhibitor and Benzonase Nuclease were added to the pellet and the pellet resuspended and incubated for 10 min with agitation at 4°C. The suspension was centrifuged at 7000×*g* for 10 min at 4°C and the supernatant (Fraction III, nuclear) collected on ice. The pellet was resuspended with extraction buffer IV with protease inhibitor cocktail (Fraction IV, cytoskeletal). Protein concentration was determined by the Non-interfering Protein Assay Kit (Calbiochem).

### Western analysis

Hearts from WT and ecSOD Tg mice were isolated, frozen, pulverized, and homogenized in buffer and homogenates prepared and processed for PAGE and Western analysis as described previously (Hu et al., 2007). Blots were probed with anti-ecSOD (1:1000, Sigma Aldrich, S4946), anti-CytC (1:1000, Abcam, 7H8.2C12), anti-vimentin (1:2000, Sigma Aldrich, V6389), Histone H3 (1:1000, Cell Signaling, 9715), and anti-E-cadherin (1:1000, Cell Signaling, 4065) antibodies, visualized by enhanced chemiluminescence and quantitated either by densitometry of film or by phosphorimager (GE, Typhoon) and analysis with ImageQuant (GE).

### Immunofluorescent confocal microscopy

ecSOD was detected and colocalized to the mitochondrial via the electron transport protein, cytochrome C (CytC) in 4 μm sections cut from paraffin embedded ecSOD Tg hearts by immunofluorescent confocal microscopy (Zeiss LSM 510). Sections were stained with anti-ecSOD (1:100, Sigma Aldrich, S4946) and counterstained with CytC (1:100, Abcam, 7H8.2C12) for mitochondrial colocalization and DAPI for nuclear staining. Line analysis of ecSOD and CytC channel intensity in confocal images was used to provide further delineation of ecSOD and CytC colocalization using NIH ImageJ(1.48Ver).

### Statistical analysis

Data are presented as the mean ± SEM. Groups were compared by unpaired Student's *t*-test where a *P* < 0.05 was considered significant.

## Results

### ecSOD Tg cardiac myocytes are resistant to hypoxia/reoxygenation injury

**Figure 1:**
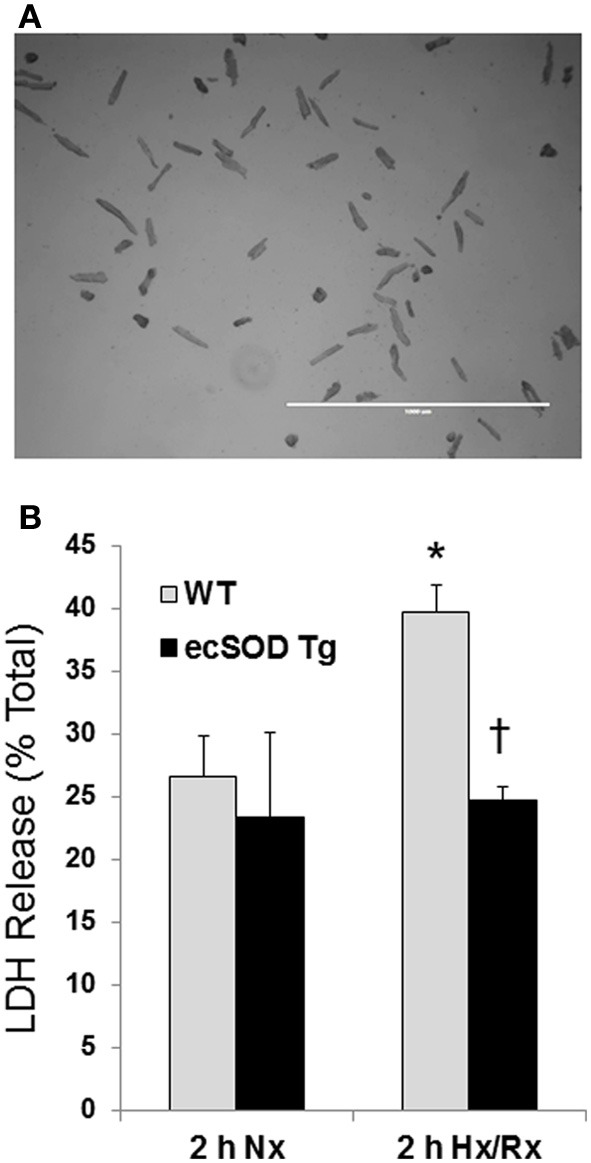
**ecSOD Tg cardiac myocytes are resistant to hypoxia/reoxygenation injury.** Cardiac myocytes from WT and ecSOD Tg mice were isolated, cultured, and incubated under hypoxic conditions, 1% O_2_, 94% N_2_, and 5% CO_2_ in hypoxic media for 30 min followed by 120 min reoxygenation (**Upper panel**, scale bar = 1 mm). Myocyte viability was assessed by measuring LDH activity in supernatants collected from cells under normoxia and after reoxygenation (**Lower panel**). Values are the mean ± SEM of 3 experiments performed in duplicate. ^*^p < 0.05 vs. Nx (WT or Tg), ^†^*p* < 0.05 vs. WT 2 h Hx/Rx.

Our previous studies have shown that cardiac specific ecSOD overexpression protects the heart from ischemia reperfusion injury supporting earlier studies showing ecSOD is cardioprotective (Obal et al., 2012). Cardiac-specific ecSOD expression also demonstrated that ecSOD could be detected in the intracellular compartment of cardiac myocytes in addition to its prototypic extracellular localization. To further investigate the action of intracellular localization of ecSOD, the effect of overexpression on hypoxia reoxygenation injury was examined in myocytes isolated from ecSOD Tg and WT mice. Myocytes, enzymatically isolated from Tg and WT hearts, were incubated under ambient oxygen conditions for 30 min after isolation. Myocytes were then incubated under 1% O_2_, 94% N_2_, and 5% CO_2_ in hypoxic medium for 30 min followed by 2 h reoxygenation under ambient oxygen with 5% CO_2_. Supernatants from WT and ecSOD Tg myocytes were collected to determine LDH release after normoxic incubation and after 120 min reoxygenation. Under normoxic conditions little difference was seen in LDH release between WT and ecSOD Tg myocytes (Figure 1). After hypoxia and reoxygenation, LDH release was significantly elevated in WT myocytes whereas release was unchanged in ecSOD Tg myocytes compared to WT (Figure 1). These results confirm our earlier results and demonstrate that the resistance to hypoxia reoxygenation injury in ecSOD Tg hearts is myocyte, autonomous.

### Mitochondrial localization of ecSOD

**Figure 2:**
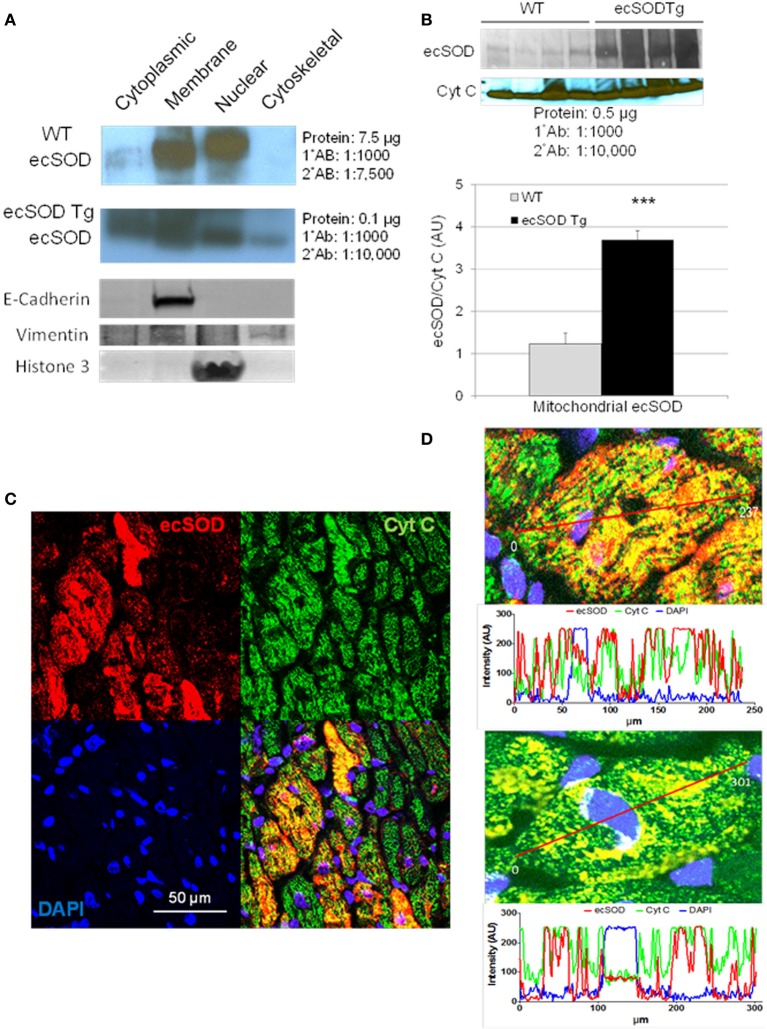
**ecSOD is expressed in intracellular compartments.** ecSOD expression was determined in membrane, cytosolic, cytoskeletal, and nuclear fractions from WT and ecSOD Tg myocytes **(A)**. ecSOD was further colocalized to mitochondria isolated from WT and ecSOD Tg hearts by Western analysis **(B)**. ecSOD colocalization with mitochondrial CytC in sections from ecSOD Tg hearts by immunofluorescent confocal microscopy **(C)**. Colocalization of ecSOD with mitochondrial CytC was demonstrated by line analysis of ecSOD and CytC emission signals from confocal images **(D)** ^***^p < 0.001 vs. WT.

Based on the intracellular localization of ecSOD highlighted by myocyte-specific transgenic overexpression, we further investigated the intracellular localization of ecSOD by subcellular fractionation and determination of ecSOD distribution by Western analysis. WT and ecSOD Tg hearts were processed for isolation of cytosolic, membrane, nuclear, and cytoskeletal fractions. Fractions from WT mice resolved by SDS PAGE and detection by Western analysis showed that ecSOD is mainly associated with the membrane fraction but was also seen at a similar level in the nuclear fraction and to a lesser degree in the cytosol (Cytosol:Membrane:Nuclear: 10%:45%:45%) and was not detectable in the cytoskeletal fraction (Figure 2A). ecSOD was markedly increased in Tg mice in all fractions. ecSOD was also shown to be associated with mitochondria isolated from both WT and ecSOD Tg hearts (Figure 2B). Notably, ecSOD levels could be detected in the mitochondrial fraction in WT mice and these levels were again markedly increased in ecSOD Tg mice compared to WT (Figure 2B). Mitochondrial localization was further examined in sections of ecSOD Tg hearts by colocalization with the mitochondrial transport chain protein, cytochrome C oxidase (CytC) by immunofluorescent confocal microscopy. In Figure 2C, intracellular ecSOD colocalizes extensively with CytC. In line analysis of ecSOD and CytC channel output in representative confocal images of myocytes stained for ecSOD (red), CytC (green), and DNA (blue), registration between ecSOD and CytC channels is very strong (Figure 2D). A fraction of cytosolic ecSOD is also found not to be associated with CytC. These results validate subcellular fraction results which demonstrate ecSOD in transgenic hearts may be found in cytosolic and nuclear (perinuclear) fractions. These results demonstrate ecSOD is capable of cytoplasmic localization with the ability to associate with intracellular organelles, specifically mitochondria.

### Increased resistance to ca^2+^ induced swelling in ecSOD Tg mitochondria

**Figure 3:**
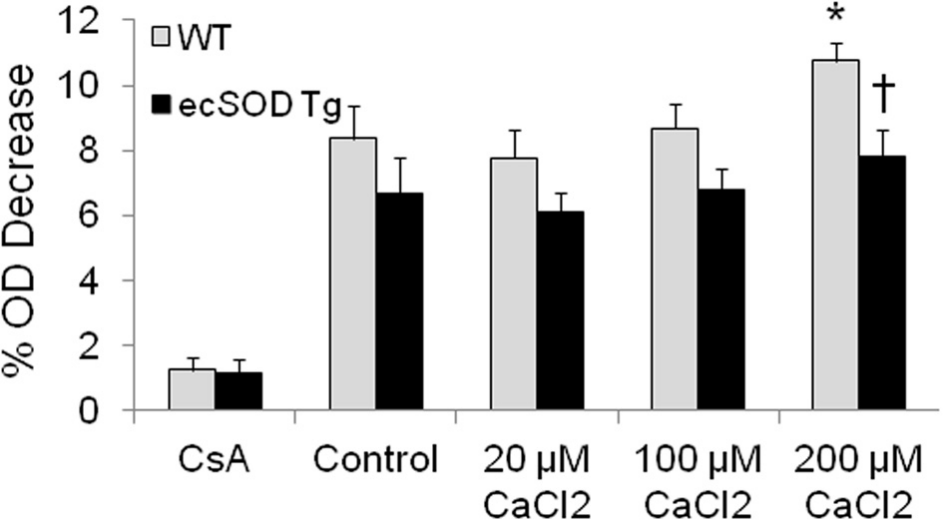
**Mitochondrial ecSOD contributes to mitochondrial permeability transition.** Mitochondria isolated from WT and ecSOD Tg hearts were incubated with control (0 μ M Ca^2+^), 20, 100, and 200 μ M Ca^2+^ to trigger MPT. MPT was measured as the percent decrease in optical density (OD) at 592 nm due to mitochondrial swelling. Mitochondrial swelling was increased with increasing Ca^2+^. Cyclosporine A (10 μM), which stabilizes mitochondrial permeability transition pore, blocked Ca^2+^-mediated swelling. Values are the mean ± SEM of 3 experiments. ^*^p < 0.05 vs. Control (WT or Tg), ^†^*p* < 0.05 vs. WT.

The enrichment of mitochondrial ecSOD in Tg mice provides an additional mechanism that can protect the myocardium from ischemia reperfusion injury. The localization of ecSOD to intracellular fractions and mitochondrial and increased resistance of ecSOD Tg myocytes to hypoxia reoxygenation injury as described above suggest alternate mechanisms may account for this action. As ecSOD was found to be specifically associated with isolated mitochondria, the effect of this association on MPT was tested by examining Ca^2+^-induced mitochondrial swelling in WT and ecSOD Tg hearts. To investigate the role of increased mitochondrial ecSOD, mitochondria were isolated from WT and ecSOD Tg hearts and swelling was measured as an index of Ca^2+^ induced MPT. Under basal conditions, WT and ecSOD Tg mitochondria exhibited similar and stable baseline OD in the presence of CsA, which binds cyclophilin D to block pore opening (Figure 3). Under control conditions in the absence of Ca^2+^, MPT was greater in WT than in ecSOD Tg mitochondria but did not reach statistical significance. With increasing Ca^2+^, WT mitochondria displayed significantly increased swelling and concomitant increased MPT. In ecSOD mitochondria, swelling was consistently and significantly less compared to WT indicating attenuated MPT. The heterogeneity of transgenic ecSOD expression in myocytes as seen in confocal microscopy images (Figure 2C) would suggest that homogeneous ecSOD expression would further accentuate the prevention of MPT in ecSOD mitochondria. These results demonstrate that MPT in ecSOD Tg mitochondria with increasing Ca^2+^ is attenuated and provides a mechanism by which transgenic ecSOD protects myocytes from hypoxia reoxygenation injury.

## Discussion

In defining the role of ecSOD in protecting the myocardium from ischemia reperfusion injury we extend our current understanding of its actions using the cardiac specific ecSOD Tg mouse. Here we demonstrate two important findings, first, that ecSOD is expressed within the myocyte intracellular compartment with increased expression seen in membrane, cytoplasm, and nuclear fractions in addition to the cytoskeletal fraction, although to a lesser degree. Localization of ecSOD expression is further narrowed to mitochondria, where, in ecSOD Tg mice, it is increased. Second, the increase in ecSOD associated with mitochondria in the ecSOD Tg mouse was able to attenuate MPT in response to Ca^2+^ challenge. These findings are important as they demonstrate that ecSOD is also expressed within the myocyte cytoplasm, is associated with mitochondria conferring resistance to Ca^2+^-induced injury through stabilization of MPT, and can contribute to protection from ischemia reperfusion injury.

ecSOD, as its name infers, has characteristically been found in the extracellular space. Previous studies have shown ecSOD nuclear translocation with oxidative stress and intracellular distribution in neurons (Oury et al., 1999; Ookawara et al., 2002). We have shown recently that myocyte-specific overexpression is able to confer myocardial protection against ischemia reperfusion injury which is associated with increased NO bioavailability (Obal et al., 2012). The intracellular location of ecSOD in myocytes demonstrated here will have significant impact on ROS and NO levels that affect cell function. ROS are generated from several intracellular locations during normal function, dysfunction, and disease. The intracellular location of ecSOD will protect myocytes from intracellular ROS induced damage in addition to extracellular ROS. We have seen that despite a relatively small contribution to total SOD activity (13%) in ecSOD Tg hearts, ecSOD has a significant survival effect (Obal et al., 2012). Mn and Cu,Zn-SOD constitute greater than 90% of activity and MnSOD has been found to be upregulated with PC and to contribute to protection and its deletion leads to postnatal lethality with cardiomyopathy (Li et al., 1995). MnSOD is predominantly localized to the mitochondrial cytosol (Slot et al., 1986). Although we have not defined the relationship with which ecSOD associates with mitochondria, association with the mitochondrial outer membrane may provide an important alternate mechanism to diminish ROS induced mitochondrial dysfunction or cell death. Mitochondrial MPT experiments are instructive, as even in the absence of Ca^2+^ MPT tended to be higher in the WT compared to ecSOD Tg and with Ca^2+^ this relationship is further exacerbated. This suggests ecSOD has a significant impact on MPT and consequential apoptotic signaling. In addition to mitochondrial localization of ecSOD in transgenic hearts, it is also found in the cytosol clearly separated from mitochondria. This may suggest ecSOD affects other intracellular functions in addition to those of the mitochondria. In the conventional ecSOD KO, deletion exacerbated myocardial dysfunction after MI and doxorubicin (van Deel et al., 2008; Kliment et al., 2009). After MI in the ecSOD KO, LV dysfunction was associated with increased nitrotyrosine which is a consequence of peroxynitrite. The decrease in peroxinitrite we have shown in the ecSOD Tg would contribute to improved function which can be associated with preserved mitochondrial function (Obal et al., 2012). As mitochondria are key contributors in ROS production with ischemia reperfusion, targeting ecSOD to mitochondria may serve as a relevant therapeutic goal.

Both constitutively expressed NOS isoforms, eNOS, and nNOS, play a role in myocyte physiology and pathophysiology (Hare, 2003). Intracellular localization of NOS isoforms and ecSOD can play a significant role in NO regulation. Localization of NOS to mitochondria has been established, however, whether one of the two isoforms or both colocalize is still controversial (Schulz et al., 2005). Originally, a mitochondrial-specific isoform, mtNOS, was proposed which has now been supplanted by either nNOS or eNOS mitochondrial localization. Regardless, with the presence of a NOS isoform in close proximity to mitochondria, ecSOD, which would be expected to be present in the extramitochondrial space, would serve to both diminish superoxide contributing to increased NO stability and bioactivity. We have shown that increased mitochondrial NO through myocardial- specific iNOS expression is able to prevent MPT in response to ischemia/reperfusion (West et al., 2008). Here we were able to show MPT was attenuated by decreased [^3^H] DOG uptake, loss of NAD^+^, and cytochrome C release. Importantly, we showed NO was able to directly prevent Ca^2+^ induced MPT. This effect of NO could be due to either NO attenuation of metabolism which would lead to decreased ROS (Trochu et al., 2000) or a direct effect on oxidation (O'Donnell et al., 1997) and these effects would be enhanced in the ecSOD Tg heart as indicated with attenuated MPT in the above discussion.

The findings presented in this study support a novel role for ecSOD in cardiac myocytes. The cardiac-specific overexpression of ecSOD demonstrates that this enzyme is capable of localizing to the intracellular space, and, in this report, with mitochondria. Myocyte-specific ecSOD is shown to be capable of providing protection from hypoxia reoxygenation; a myocyte autonomous effect. Mitochondria from ecSOD Tg mice are resistant to Ca^2+^-induced MPT that constitutes a mechanism by which it can contribute to myocyte survival from the intracellular space. In contrast to MnSOD or CuZnSOD that may be constrained by localization to the mitochondrial matrix, ecSOD can expand its antioxidant capabilities by boosting intracellular expression. These insights are important in understanding ecSOD's contributions to protecting the myocardium and its potential as a therapeutic such as that in gene therapy (Li et al., 1998, 2001; Qi et al., 2007a,b; Deng et al., 2010; Saqib et al., 2011).

### Conflict of interest statement

The authors declare that the research was conducted in the absence of any commercial or financial relationships that could be construed as a potential conflict of interest.
